# Krill Products: An Overview of Animal Studies

**DOI:** 10.3390/nu7053300

**Published:** 2015-05-07

**Authors:** Lena Burri, Line Johnsen

**Affiliations:** Aker BioMarine Antarctic AS, Fjordalléen 16, NO-0115 Oslo, Norway; E-Mail: line.johnsen@akerbiomarine.com

**Keywords:** dietary supplement, krill oil, krill powder, omega-3 phospholipids, animal studies

## Abstract

Many animal studies have been performed with krill oil (KO) and this review aims to summarize their findings and give insight into the mechanism of action of KO. Animal models that have been used in studies with KO include obesity, depression, myocardial infarction, chronic low-grade and ulcerative inflammation and are described in detail. Moreover, studies with KO in the form of krill powder (KP) and krill protein concentrate (KPC) as a mix of lipids and proteins are mentioned and compared to the effects of KO. In addition, differences in tissue uptake of the long-chain omega-3 polyunsaturated fatty acids eicosapentaenoic acid (EPA) and docosahexaenoic acid (DHA), when delivered in either phospholipid or triglyceride form, are addressed and the differential impact the delivery form has on gene expression profiles is explained. In our outlook, we try to highlight the potential of KO and KP supplementation in clinical settings and discuss health segments that have a high potential of showing krill product specific health benefits and warrant further clinical investigations.

## 1. Introduction

Krill are small crustaceans particularly abundant in Northern (Arctic) and Southern (Antarctic) polar seas. The oil is extracted from the largest of the krill species, the Antarctic *Euphausia superba*. Krill is a sustainable source of omega-3 polyunsaturated fatty acids (*n*-3 PUFAs) and the oil is especially rich in the long-chain PUFAs eicosapentaenoic acid (EPA; C20:5 *n*-3) and docosahexaenoic acid (DHA; C22:6 *n*-3). While fish oil (FO) mainly contains triacylglyceride (TAG)-bound *n*-3 PUFAs, krill oil (KO) contains a major part of these fatty acids (FAs) bound in phospholipids (PLs) with phosphatidylcholine (PC) being the most abundant form [1,2]. More than 80% of EPA and DHA in KO are found in the PC form (Table 1).

**Table 1:** Krill oil (KO), krill powder (KP) and krill protein concentrate (KPC) compositions—Typical values.

Composition	KO (g/100 g Oil)	KP (g/100 g Powder or Extracted Fat)	KPC (g/100 g KPC or Extracted Fat)
**Protein**	-	38	78
**Total lipids**	89	51	8
**Triglycerides**	34	45 *	-
**Total PLs**	43	43 *	-
**PC**	35	40 *	-
**Total omega-3**	25	20 *	27 *
**EPA**	13	10 *	12 *
**DHA**	7	5 *	13 *
**Total omega-6**	2	2 *	4 *
**Saturated FA**	23	25 *	37 *
**Total MUFAs**	15	20 *	21 *

* Values are given as g/100g extracted fat.

Supplementation with KO gives the advantage of not only supplying EPA and DHA, but also choline, which is a conditionally essential nutrient [3]. Choline is needed in the synthesis of neurotransmitters (acetylcholine) and PLs (PC, lyso-PC, choline plasmalogen and sphingomyelin) and is important in the transport of lipids and reduction of homocysteine. The human body can synthesize choline *de novo*, but this is not sufficient to meet all the body’s requirements and the rest needs to be taken up from the diet. Although most diets are considered to provide sufficient choline, populations vulnerable to choline deficiency exist. Moreover, the National Health and Nutrition Examination Survey in 2003–2004 has reported an inadequate intake of choline in 90% of the American population [4]. Choline deficiency is linked to an increased risk for fatty liver and muscle dysfunction [5] and *n*-3 PC supplementation could be beneficial to people that are not reaching an adequate intake of choline from their diet to ensure optimal membrane function, neurotransmission and methyl donor availability.

Moreover, there is increasing evidence that PLs might be a more effective delivery form of *n*-3 PUFAs to body tissues than TAGs [6,7,8,9]. DHA accounts for 40% of the FAs in the human brain and accretion of it in the brain has been of particular interest [10]. Single-dose studies with radiolabelled DHA have found an about two-fold higher tracer brain accumulation, when DHA was provided in PL rather than TAG form [6,7]. Interestingly, the transporter for DHA into the brain has recently been identified [11] and it was shown that DHA is transported in lyso-PC form and not as unesterified FA [11,12].

While the long-chain PUFAs, EPA and DHA, are derived from marine organisms, short-chain *n*-3 PUFAs can be found in vegetable sources or non-marine animal sources [13]. Only one of the *n*-3 FAs is found in plant-based sources, *i.e.*, α-linolenic acid (ALA 18:3; *n*-1). However, in humans only a small amount of ALA is converted to EPA, and the conversion of ALA to DHA is even less efficient and estimated to be under 1% [14]. Consequently, dietary intake of EPA and DHA might help to optimize human health.

A substantial amount of studies on EPA and DHA have assessed the health benefits of *n*-3 PUFAs [15,16] and have found them to be crucial at cellular levels for maintaining membrane homeostasis, as ligands for transcription factors thus influencing gene expression, and for an optimal balance with omega-6 FAs for the formation of eicosanoids and endocannabinoids. Through these means they have the potential to influence regulation of inflammatory response, neurological and psychiatric balance and body composition homeostasis [17,18,19].

Most *n*-3 studies have been performed by using *n*-3 PUFAs in TAGs or ethyl esters. However, more recently, KO rich in *n*-3 PUFAs bound in PLs has received increasing attention in several animal and some clinical studies. This review aims to give an overview over the KO animal studies only, while the clinical studies will be addressed elsewhere.

## 2. Documentation of Krill Oil Health Benefits in Animal Studies

A subchronic toxicity study and genotoxicity studies in rats have been done to confirm the safety of KO and the no observed adverse effect level (NOAEL) for this study was considered to be 5% KO [20]. Subsequent animal studies with KO have included a wide range of models such as ulcerative colitis [21], depression [22], obesity [23,24], and myocardial infarction in rats [25], or tumor necrosis factor-alpha (TNFα)-overexpression [26], rheumatoid arthritis [27], and dietary obesity in mice [28,29,30,31]. Additionally, one study in castrated rabbits has investigated glucose tolerance [32] (Table 2).

**Table 2:** Overview of the main animal studies with krill oil (KO), krill powder (KP) and krill protein concentrate (KPC) that are discussed in the selected health areas. Only one high dose study shows adverse events [33].

Classification of health benefits	Study	Animal model	Diets	Duration of Supplementation	Effect of KO/KPC and KP
					Effect of KO
Obesity	Zhu *et al.*, 2008 [34]	SD rats (obesity model) *n* = 60	Different doses: High fat with 16.7, 33.3, 99.9 or 199.8 g/L KO	4 weeks	decreased levels of serum TAG, TC and LDL-C
					reduced body weight
Obesity	Batetta *et al.*, 2009 [23]	Zucker rats (obesity model) *n* = 18	Control diet with 0.5 g EPA+DHA/100 g diet (from either KO or FO)	4 weeks	decreased levels of plasma LDL-C
					increased levels of plasma TAG
					reduced TAG in liver and heart
					decreased AEA and 2-AG in VAT and AEA in liver and heart
					reduced TNFα secretion from macrophages (associated with lower levels of AA in membrane PLs)
Obesity	Tandy *et al.*, 2009 [30]	C57BL/6 mice (obesity model) *n* = 46	Different doses: Normal diet or high fat diet with 1.25%, 2.5% or 5.0% KO	8 weeks	reduced hepatic steatosis and plasma glucose and TC (not TAG)
					increased adiponectin
					reduced hepatic TNFα expression and down-regulation of several hepatic genes involved in FA synthesis and catabolism
Obesity	Di Marzo *et al.*, 2010 [28]	Zucker rats (obesity model) *n* = 18	Control diet with 0.5 g EPA+DHA/100 g diet (from either KO or FO)	4 weeks	increased EPA and DHA levels in brain
					decreased 2-AG levels in brain
Obesity	Piscitelli *et al.*, 2011 [29]	C57BL/6 mice (obesity model) *n* = 6–10 per group	Different doses: Normal diet or high fat diet with 1.25, 2.5 or 5% KO	8 weeks	reduction of AEA and/or 2-AG levels in heart, kidneys, gastrocnemius muscle, inguinal and epididymal adipose tissue
Obesity	Ferramosca *et al.*, 2012 [24]	SD rats *n* = 120	Control, High fat control, High fat 2.5% KO	12 weeks	decreased levels of hepatic TAG and cholesterol
					reduced plasma TAG and glucose levels
					reduced hepatic FA synthesis
					increased hepatic FA oxidation
					increased mitochondrial respiration efficiency
					reduction in body weight
Obesity	Tillander *et al.*, 2014 [31]	C57BL/6J mice	High fat control (*n* = 9) High fat FO (5.8%) (*n* = 6) High fat KO (5.7%) (*n* = 6)	6 weeks	decreased plasma levels of NEFA
					down regulation of cholesterol and fatty acid synthesis (mRNA level)
Obesity	Ivanova *et al.*, 2014 [32]	New Zealand white rabbits *n* = 24	Castrated control, Non-castrated control, Castrated KO (daily dose of 600 mg omega-3), Castrated FO (daily dose of 600 mg omega-3)	2 months	decreased fasting glucose for both FO and KO
					modified gene expression of key enzymes in β-oxidation, lipogenesis in liver and skeletal muscle
Inflammation	Ierna *et al.*, 2010 [27]	DBA/1 mice (arthritis model) *n* = 42	Control diet with 0.44 g EPA+DHA/100 g diet from KO or 0.47 g EPA+DHA/100 g diet from FO). Induction of arthritis Day 25, boost Day 47	68 days	reduction in paw swelling
					reduction in histopathology scores (joint section)
Inflammation	Grimstad *et al.*, 2012 [21]	Wistar rats (colitis model) *n* = 30	Control, Control + DSS, Control + DSS + KO, Induction of colitis by DSS on Day 23	30 days	improved colon length
					increased level of (PG)E_3_ and Pparg1 α expression
Cardiovascular	Fosshaug *et al.*, 2011 [25]	Wistar rats (myocardial infarction model)	Control n = 14 pre-treated (2 weeks) with KO *n* = 18 not pre-treated with KO *n* = 17 Induction of myocardial infarction by left coronary artery ligation	7 weeks	Pre-treated KO group: - attenuated LV dilation - reduced heart and lung weights - reduced mRNA levels of LV stress and matrix remodeling markers
					Not pre-treated KO group: - increased LV
Brain	Gamoh *et al.*, 2011 [35]	Wistar rats	Control (n = 15) High dose krill PL (420 mg EPA+DHA) (*n* = 14) Low dose krill PL (301 mg EPA+DHA) (*n* = 13)	6 weeks	improved spatial-memory related learning ability
					increased levels of EPA, DPA and DHA and decreased level of AA in the brain
					decreased levels of lipid peroxide and reactive oxygen species
					increased cell generation in dentate gyrus
Brain	Wibrand *et al.*, 2013 [22]	Wistar rats	Control (*n* = 12) Imipramine (*n* = 12) KO 1.25% (*n* = 14) KO 2.50% (*n* = 14)	7 weeks	improved learning and memory processes
					anti-depressant-like effects
Liver	Ferramosca *et al.*, 2012 [36]	Wistar rats *n* = 18	Control, 2.5% KO, 2.5% FO	6 weeks	decreased levels of plasma TAG and TC
					inhibition of hepatic lipogenesis (reduced activity of CIC, ACC and FAS)
Gene expression	Burri *et al.*, 2011 [37]	CBA/J mice*n* = 30	Control diet with 1.5% KO or 1.1% FO	3 months	down-regulation of: hepatic glucose pathways, lipid and cholesterol synthesis (FO up-regulated cholesterol synthesis pathway)
Kidney	Gigliotti *et al.*, 2013 [33]	Female SD rats *n* = 60	12% corn oil (*n* = 10) 12% flaxseed (*n* = 10) 12% menhaden oil (*n* = 10) 12% KO (*n* = 10) 12% salmon oil (*n* = 10) 12% tuna oil (*n* = 10)	8 weeks	increased kidney weight
					increased calcium content of the kidneys
					increased urinary phosphorous excretion
Safety	Robertson *et al.*, 2014 [20]	Wistar rats *n* = 80	Control, 1.7% KO, 3.3% KO, 5% KO	13 weeks	NOAEL is 5% KO
					Effect of KPC and KP
Inflammation	Bjørndal *et al.*, 2012 [38]	C57BL/6 mice constitutively expressing hTNFα gene (*n* = 20)	High fat control; High fat 3% KP	6 weeks	decreased plasma and liver TAG levels
					down-regulation of hepatic genes involved in lipogenesis
					reduction of TNFα in liver
Kidney	Gigliotti *et al.*, 2008 [40]	Female SD rats *n* = 30	10% KPC, 10% casein	4 weeks	reduced kidney weight
					reduced total mineral content of the kidneys
					no differences in kidney function
Kidney, Bone	Gigliotti *et al.*, 2011 [41]	Female SD rats *n* = 20	10% KPC, 10% casein	4 weeks	reduced kidney injury (lower urinary NAG activity, reduced kidney mineralization, tendency for higher GRFs and lower proteinuria) and Ca deposition
					no effect on bone mass or strength
Gene expression	Bjørndal *et al.*, 2013 [41]	CBA/J mice *n* = 20	Low fat control Low fat 3% KP	3 months	large number of pathways are modulated
					down regulated pathways: β-oxidation, glucose metabolism and amino acid catabolism
Safety	Bridges *et al.*, 2010 [42]	Female SD rats *n* = 20	10% KPC, 10% casein	4 weeks	increased DHA concentration in brain, and increased EPA and DHA concentration in fat pads and liver
					decreased pro-inflammatory 2-series prostaglandin and thromboxan metabolites
Safety	Berge *et al.*, 2014 [43]	Wistar rats	Control, 9.67% KP	13 weeks	NOAEL is 9.67% KP

Abbreviations: AA, arachidonic acid; ACC, cytosolic acetyl-CoA carboxylase; AEA, N-arachidonoylethanolamine; 2-AG, 2-Arachidonoylglycerol; DHA, docosahexaenoic acid; DSS, dextran sulfate sodium; CIC, mitochondrial citrate carrier; EPA, eicosapentaenoic acid; FAS, fatty acid synthetase; FO, fish oil; GRF, glomerular filtration rate; KO, krill oil; KP, krill powder; KPC, krill protein concentrate; LDL-C, low density lipoprotein cholesterol; LV, left ventricular; NAG, n-acetyl glucosaminidase; NEFA, non-esterified fatty acids; NOAEL, no observed adverse effect level; PL, phospholipid; SD, Sprague Dawley; TC, total cholesterol; TAG, triglyceride; VAT, visceral adipose tissue.

### 2.1. Different tissue FA Distribution When Carried in PL Versus TAG Ester Forms

In some studies, the effects of *n*-3 PUFAs from KO were more pronounced than the ones observed with FO [44,45,46]. This may be due to the fact that KO contains EPA and DHA mainly in the chemical form of PLs with the attributed increased tissue delivery efficacy [6,7,9].

For example, in a study with obese Zucker rats, it was shown that EPA and DHA had higher incorporation into heart, when they are provided as KO rather than FO [23]. Hence, after providing FO or KO supplemented diets balanced to a final EPA and DHA concentration of 0.5g/100g for 4 weeks, the heart PL FA profile showed 97 and 42% higher concentrations of EPA and DHA, respectively, for the KO compared to the FO supplemented group. A similar trend was observed in the liver, were the corresponding incorporation of EPA and DHA into PLs was 47% and 13% higher after KO supplementation.

In brain PLs, significant higher EPA and DHA levels were found after 4 weeks of feeding KO compared to FO supplementation (0.5 g of EPA and DHA per 100 g of diet), *i.e.*, 77 and 24% higher levels, respectively [28]. These findings are in line with other studies in rats, baboons and pigs comparing single radiolabeled FA doses in either PL or TAG forms [6,7,9].

Overall, this may indicate that the dietary oil (FO *versus* KO) and therefore the FA delivery form (TAG *versus* PL) may influence EPA and DHA incorporation into brain lipids.

### 2.2. Krill Oil—Endocannabinoid System

The endocannabinoid system, consisting of endogenous cannabinoids called endocannabinoids (ECs) and their receptors, is involved in a variety of physiological processes. If dysregulated it might be implicated in different pathological states, such as metabolic syndrome and cardiovascular and mental diseases [47,48]. In obese subjects it was shown that the ECs called *N*-arachidonoyl-ethanolamine (AEA; anandamide) and 2-arachidonoylglycerol (2-AG) are elevated in the blood. They are made by enzymatic reactions from arachidonic acid (ARA) depending on its position in PLs (AEA when in 1-position and 2-AG when in 2-position). Hence, the more omega-6 ARA is available in PLs, the more ECs can be made. EPA and DHA however will compete for the esterification into PLs and an increased intake of *n*-3 PUFAs might help to counterbalance a disturbed *n*-3 to *n*-6 ratio and result in lower EC levels that may positively affect membrane signaling and energy metabolism [49].

In studies with Zucker rats, a model for obesity and its related metabolic dysfunctions, KO has been shown to influence EC levels both in peripheral tissues, as well as in the brain [8,23,28]. After 4 weeks with a low dose KO supplementation, the AEA and 2-AG levels were reduced around the organs in visceral adipose tissue compared to control, while no differences were observed beneath the skin in subcutaneous adipose tissue. In both heart and liver, KO supplementation lead to a significant decrease in AEA, but not 2-AG levels, as compared to control. Moreover, significant reductions in TAG levels in liver and heart were also observed after KO supplementation [23]. These findings strengthen previous observations that AEA is responsible for TAG deposition in rodents [50]. In addition to decreased TAG levels in heart and liver, a 75% lower plasma LDL-cholesterol concentration was found in KO supplemented rats compared to controls [23]. EC receptor over-activity has previously been associated with increased fat accumulation in visceral adipose tissue in rodents and in humans. However, in spite of the reduced levels of ECs, no effect on body weight could be observed in this short-term experiment.

Besides their important role in metabolic processes, ECs are also considered to affect brain function. The EC receptors in the central nervous system (CNS) have been shown to influence the control of emotional responses to stress and anxiety. While a blockade of EC receptors in peripheral tissue reduces food intake and body weight, a prolonged attenuation of these receptors in the brain were found to have severe neuropsychiatric side effects [51]. To further investigate how KO influences the EC profile in the brain, levels of various ECs were measured in obese Zucker rats after 4 weeks of feeding with KO [28]. The results revealed that while 2-AG was significantly lower after KO administration compared to placebo and FO, levels of AEA was similar in all groups. DHA and EPA were significantly higher in brain PLs after KO administration compared to placebo and FO, whereas no difference was observed for ARA. However, no differences in growth or feed intake between the KO and control group were observed, indicating that the difference in 2-AG levels was not sufficient to exert such effects. It is worth noting that KO administration did not affect AEA levels and is therefore unlikely to negatively interfere with stress and anxiety symptoms [52].

The effect of KO on the EC system has also been investigated in other tissues, such as muscle and kidney. In a study performed by Piscitelli *et al.*, mice on a high-fat diet were supplemented with KO doses ranging from 1.25%–5% for 8 weeks [29]. While the high-fat diet increased the levels of both 2-AG and AEA in muscle as well as kidney, all doses of KO supplementation efficiently reduced these levels to even lower values than those measured for the controls. Supplementation with KO also ameliorated the increased hepatic TAG, cholesterol and TNF-α values induced by the high-fat diet. The animal studies performed so far indicate therefore that KO might have a positive effect on obesity-associated pathologies by counteracting elevated EC levels peripherally and at the same time avoiding the negative psychiatric effects observed with reduced EC activity in the brain.

### 2.3. Krill Oil—Body and Tissue Weights

A reduction in body weight as a result of KO supplementation in obesity models has been reported in some animal studies [24,34], while others did not support such an effect [28,29,30]. In one study, where a hyperlipidemic rat model was established, supplementation with KO to a high-fat diet over a 4-week period resulted in significant lower body weights compared to the controls fed only the high-fat diet [34]. In another study, rats were fed a control diet, a high-fat diet or a high-fat diet supplemented with 2.5% krill oil for 12 weeks. In the high-fat-fed rats the increase in body weight was significantly prevented by the supplementation of KO [24].

In high-fat-fed mice, KO has also been demonstrated to be effective in improving metabolic factors after 8 weeks of treatment with 3 different doses (1.25, 2.5 or 5% KO) [30]. The experiment was carried out to investigate the dose-dependent effect of KO on liver weight and a significant lower weight was observed in the KO-supplemented high-fat-fed mice compared to control mice. The difference in liver weight was associated with a significant dose-dependent decrease in total hepatic fat content.

### 2.4. Krill Oil—Glucose Tolerance

The high-fat-fed mice study mentioned above further demonstrated that neither serum glucose, insulin, nor adiponectin levels were significantly different in normal *versus* high-fat-fed-mice. However, the mice fed the high-fat diet supplemented with KO had significantly lower fasting serum glucose and higher serum adiponectin levels compared to the high-fat fed mice not receiving KO [30]. Only serum insulin levels were unaffected by KO supplementation. Adiponectin is a protein secreted by adipose tissue. It is of interest that KO has the ability to increase this anti-inflammatory and anti-atherogenic hormone, since it has the ability to stimulate insulin sensitivity by increasing FA oxidation in the liver and skeletal muscle [53].

Improved insulin sensitivity and secretion after administration of 600mg KO per day could also be seen in an obesity model of castrated male New Zealand white rabbits [32]. Expression levels of key enzymes in β-oxidation and lipogenesis were different after KO feeding for 8 weeks, compared to placebo, which ultimately led to decreased fasting blood glucose and improved glucose tolerance in the rabbits. However, when the KO administered is outside of the 5% NOAEL at a level of 11.8% of the diet, then the rats supplemented for 8 weeks showed significantly higher kidney weights, total calcium content, and renal calcification and tubulo-interstitial injury compared to the control group [33]. Authors have suggested that the reason might be the higher urinary phosphorus excretion due to the phospholipid content of KO.

### 2.5. Krill Oil—Chronic Inflammation, Rheumatoid Arthritis and Ulcerative Colitis

Chronic low-grade inflammation and obesity are associated with elevated levels of TNFα and KO has been shown to have a positive influence on inflammation in both high-fat-fed mice and in obese Zucker rats [23,26]. Compared to mice fed a normal diet, high-fat-fed mice showed significantly elevated levels of hepatic TNFα [26]. However, supplementation of a high fat diet with KO (1.25 to 5%) significantly reduced the amount of TNFα protein produced.

Inflammatory parameters were also investigated in Zucker rats treated with either FO or KO for 4 weeks [23]. The FA analysis of peritoneal macrophages showed a significant difference for both FO and KO fed rats when compared to control. EPA and DHA increased significantly in the macrophages, whereas ARA was reduced. To assess whether FA membrane composition could influence the inflammatory response in these cells, they were incubated for 24 h with lipopolysaccharide (LPS) and cytokine secretion was measured. After LPS stimulation, TNFα secretion was significantly lower in both supplemented groups in comparison to control. The dampened inflammatory response after FO and KO administration can be explained by the reduction of ARA. However, the levels of pro-inflammatory cytokines (TNF-α, IL-6, and IL-1β), anti-inflammatory cytokines (IL-10 and TGF-β) and C-reactive protein in plasma were not significantly different among the experimental groups.

In addition to the anti-inflammatory effects of KO observed in the Zucker rats, further anti-inflammatory benefits of KO treatment were seen in a collagen-induced arthritis model in DBA-1 mice [27]. Three diet groups were investigated: Control, KO, and FO with a final amount of EPA and DHA of about 0.45 g/100 g in the KO and FO groups. The mice were fed these diets for 25 days prior to induction of arthritis by an injection of collagen emulsified in adjuvant, and continued to receive treatment diets until a 10-week period was fulfilled. The first arthritis symptoms appeared 47 days from start of the study, when the mice obtained another collagen boost. Coincidental with the development of more severe arthritis, a drop in body weight was observed in all three groups. The animals fed KO, however, had significantly higher body weights compared to the control animals from day 3 until the end of the study at all days measured. At termination of the study was the average weight in the FO group 19.8 g, while in the KO group it was 23.8 g and in the control group 19.5 g.

Clinical signs of arthritis were assessed in all paws according to a 0 to 4 scale of ascending severity and paw thickness was measured. In the control group, clinical arthritis was noted prior to the collagen boost and increased steadily during the course of the experiment. In the KO group, the disease incidents increased more slowly and were not observed at all until day 47. On study day 45, when mice were first examined for signs of arthritogenic responses, the mean clinical arthritis score (mean ± SEM) was 0.2 ± 0.2 in the control group, 0.0 ± 0.0 in the FO group and 0.0 ± 0.0 in the KO group. The arthritis scores continued to increase until the end of the study, when peak scores were observed in each group. On day 68, the score was 11.3 ± 0.9 in the control group, 8.4 ± 1.0 in the FO group and 5.9 ± 1.4 in the KO group. At study termination the paw thickness in the FO group was 3.0 mm and 2.9 mm in the KO group, whereas it was 3.3 mm in the control group. Already on days 25, 28, 31 and 54 and from day 60 onwards, paw thickness measurements were significantly lower in mice fed KO compared to those in the control group. Administration of FO induced significant differences from control animals at day 49 and 54 only.

The right rear paws were further processed for histology and scored for cellular influx, synovial hyperplasia or erosion of bone/cartilage. It was found that KO was able to significantly reduce synovial hyperplasia and cellular infiltration, as well as displaying a trend towards reduced bone/cartilage erosion. Treatment with FO reduced the synovial hyperplasia and total histology score, but did not affect cell influx.

KO also appears to attenuate inflammation in an experimental model of ulcerative colitis in rats [21]. In the study, male rats were divided into three groups of 10 animals each, receiving either control, control + dextran sulfate sodium (DSS) or 5% KO + DSS diets for 30 days. DSS was added to the drinking water for the last week of the experiment to induce colitis that resembles human ulcerative colitis, a mucosal disease involving all or part of the colon.

One of the main findings was that the colon length, which was significantly shortened in the DSS-treated compared to control animals, was preserved in the rats supplemented with KO. The DSS-induced reduction in colon length is suggested to result from edema and inflammation in the colonial mucosa [54].The colon length preservation in rats supplemented with KO hence suggests a protective effect against colonial inflammation. 

The PGE_3_ eicosanoid, resulting from the metabolism of *n*-3 PUFAs, was significantly increased in the DSS + KO group *versus* the DSS group. Other factors as disease activity index (DAI) and TNF-α and IL-1β levels also tended to be positively affected by KO compared to DSS administration alone. 

To investigate the effect of KO on oxidative stress in the DSS-treated mice, several markers of protein oxidation were measured. The proteins, carbonyl glutamic semialdehyde and the glycoxidation products carboxyethyl lysine and carboxymethyl lysine, were all significantly reduced in the distal colon after KO administration.

### 2.6. Krill Oil—Cardiovascular Risk Markers and Myocardial Infarction

The main underlying cause of cardiovascular disease (CVD) is atherosclerosis, a process marked by abnormal build-ups of lipids, cholesterol and other substances in arteries [55]. In order to maintain cardiovascular health, emphasis has been put on the importance of the maintenance of normal TAG and high- and low-density lipoprotein (HDL and LDL) levels, preventing high blood pressure and thrombotic events [56].

Blood TAG and cholesterol concentrations have been found to be significantly reduced by administration of KO both in normolipidemic rats [36] and in rats where hyperlipidemia was provoked by a high-fat diet [24,34]. In the experiment with normolipidemic male Wistar rats, a standard diet, supplemented either with olive oil, 2.5% FO or 2.5% KO was fed for 6 weeks [36]. At the end of dietary treatment, the reduction in plasma TAG levels was 27% in both KO- and FO-fed animals with respect to control rats. The reduction in plasma total cholesterol was around 15% in both treatment groups.

In the experimental model of obesity, male Sprague Dawley rats were fed a high-fat diet [34]. Hyperlipidemia, decreased total cholesterol, TAG, and HDL, as well as increased LDL levels were confirmed after 2 weeks of high-fat-feeding. 5 groups of animals with different KO doses were investigated, *i.e.*, from 0 to 200 g/L KO. Significant changes in serum lipids were observed after 4 weeks of consuming KO and showed a lowering effect on TAG levels with all 4 KO doses, as well as a decrease in total cholesterol and LDL levels. The two highest KO doses (100 and 200 g/L) also significantly increased HDL levels.

More results with KO on cholesterol were observed in high-fat-fed male C57BL/6 mice supplemented with 0%, 1.25%, 2.5% and 5% of KO [30]. After 8 weeks of administration, serum cholesterol levels were significantly reduced by 20%, 29% and 29% in the 1.25%, 2.5% and 5% KO groups, respectively.

Not only can KO influence risk factors for CVD, KO was also found to have a direct effect on cardiac remodeling and function in an experimental myocardial infarction (MI) rat model [25]. In this experiment rats were randomized to pre-treatment with KO or control for 14 days before induction of MI. 7 days post-MI, the rats were examined with echocardiography and control rats continued either on control or KO feed for 7 weeks before another examination with echocardiography and euthanasia. Echocardiography showed a significant attenuation of left ventricular (LV) dilation in the group pre-treated with KO in comparison to controls. Attenuated heart and lung weights, and mRNA levels encoding classical markers of LV stress, matrix remodeling and inflammation reflected these findings.

### 2.7. Krill Oil— Cognitive Function/Depression

Emerging evidence over the last decade indicates that *n*-3 PUFAs play an important role in maintaining and enhancing functions of the CNS. However, so far only two animal studies have specifically investigated the effect of KO on cognitive function and depression [22,35].

One study indicated enhanced short- and long-term memory in rats after 6 weeks of supplementation with krill PLs [35]. Memory function (spatial learning) was assessed by performing a radial arm maze task, where reward pellets were placed on any 4 arms of the 8 arms of the maze. Two different indicators, Reference Memory Error (RME) and Working Memory Error (WME) were used to evaluate the test. RME defines long-term memory in an inverse relationship by counting how many times arms without reward-pellet were selected and WME describes as an inverse indicator the ability for short-term memory by counting the re-selected arms. During the study, each rat was given 2 trials daily, 6 days per week for a total of 3 weeks. The treatment continued through the whole maze task period. Both RME and WME significantly decreased with increased number of trials, for both the low and high dose krill PL groups compared to control, suggesting a potential for KO to improve both short- and long-term memory. In addition, increased *n*-3 PUFA and decreased ARA levels were measured both in plasma and brain after krill PL treatment. Furthermore, lipid peroxidation in plasma and brain was depressed, cell generation in the dentate gyrus was enhanced and reactive oxygen species in the cerebral cortex and hippocampus were lowered in the krill PL-supplemented rats.

A later study evaluated the effect of KO on cognition and depression-like behavior in rats [22]. Using the Aversive Light Stimulus Avoidance test (ALSAT), cognition is assessed by the rats’ abilities to separate between an active lever and an inactive lever during exposure to bright light. Whenever pressed down, the active lever causes a 30 s period of darkness, while there is no effect when pressing the inactive lever. In this 7-week study, the rats received either KO corresponding to 1.25% of the daily ration of food, the anti-depressant Imipramine (IMIP) as a positive reference substance or water as a control. On the day of testing, the KO group displayed a similar level of activity as the control group, regarding the total number of lever presses. The IMIP group, however, showed signs of sedation by reduced activity levels. In the control group, the rats pressed equally many times on the inactive and the active levers, while the rats in the KO group pressed the active lever significantly more frequent than the inactive lever. This indicates a learned ability to separate between the two levers.

Further a forced swimming test was used, where rats are exposed to an inescapable water situation. The time of passive floating or absence of movement in the water is considered as a measure for depression-like behavior. Both the group receiving KO and the group receiving IMIP, had significantly decreased immobility time as compared to the control animals, suggesting an anti-depressant effect. These results indicate that active components in KO facilitate learning and memory processes and provide anti-depressant effects.

### 2.8. Krill Oil—Gene Expression

**Figure 1:**
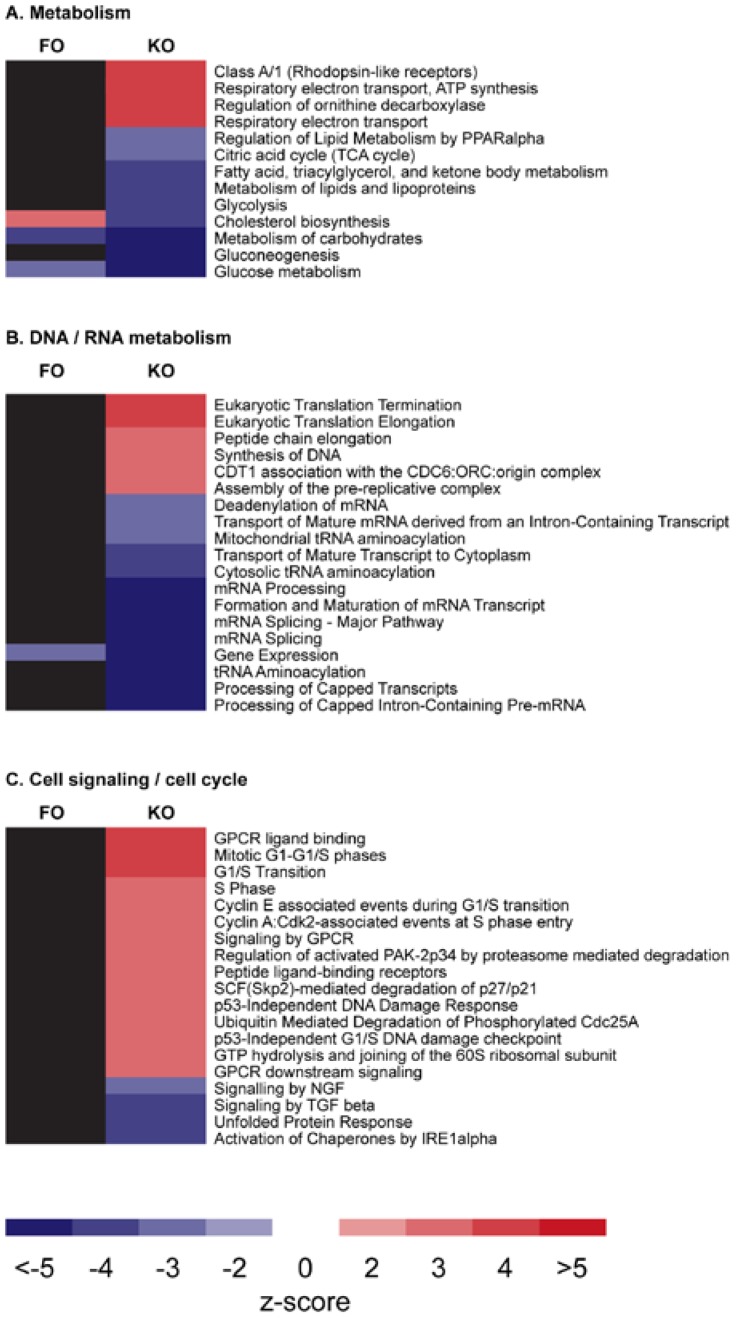
Identified hepatic metabolic pathways that are modulated after krill oil (KO) and fish oil (FO) feeding to mice. Each row indicates a separate pathway from the Reactome.org database. The first column indicates the effect of FO, the second one the effect of KO. Red cells are pathways that are significantly upregulated by treatment (either FO or KO). If the cell is blue, then the pathway was significantly downregulated by treatment (either FO or KO). A black fill indicates that the pathway activity was not changed by treatment. The figure was kindly contributed and adapted by J. Barger from [37] with permission of the authors.

In order to investigate the effects of KO on gene expression and molecular pathways, several animal studies have been performed [24,30,31,36,37]. The ability of EPA and DHA from KO to influence gene expression by binding to specific gene transcription factors was explored in a 12-week study on healthy CBA/J mice [37]. The mice were fed a low-fat diet supplemented with either KO or FO that contained similar EPA and DHA concentrations in the diet. Even though both diets contained approximately 0.3% of EPA and DHA in a similar ratio, the *n*-3 PUFAs from the two different sources, when compared to a control, affected a different number of molecular pathways; while KO modulated 52, FO only affected 4 pathways. The 4 pathways changed by FO were also altered by KO, however, whereas the activity of the pathway ‘Cholesterol biosynthesis’ was higher in the FO mice, it was lower in the KO group. Moreover, pathway analysis after KO supplementation suggested that glucose, FA and lipid metabolisms were positively affected and energy production by mitochondria was higher (Figure 1). Not the same biological response could be seen in the FO-receiving mice and an explanation for this difference may be the different *n*-3 lipid structures of either PLs in KO *versus* TAGs in FO that can lead to different hepatic membrane integration efficiencies and hence *n*-3 availability for transcription factor binding.

The results obtained from this study were also confirmed in a study with high-fat-fed rats, where the effects of 1-6 weeks of 2.5% KO or FO supplementation on enzyme activities in hepatic lipogenesis was compared [24]. Inasmuch, the results showed that KO reduced the protein levels and enzyme activities involved in fat generation. In particular, the activity of the tricarboxylate carrier (mitochondrial carbon unit transport carrier involved in lipogenesis) has been found to be decreased and more so than in the FO supplemented livers at shorter feeding times. In addition, activities of enzymes involved in FA synthesis, *i.e.*, acetyl-CoA carboxylase and FA synthetase were decreased. Simultaneously, hepatic FA oxidation was increased, shown by increased activity of carnitine palmitoyltransferase I and carnitine levels. Both KO and FO treatment led to similar reductions in plasma cholesterol and TAG, whereas the livers of KO supplemented animals revealed a stronger reduction in these parameters than the FO treated animals. Moreover, KO supplementation counteracted the negative effects of a high-fat diet by stabilizing oxidative damage to lipids and proteins, preventing body weight increase and maintaining an efficient mitochondrial respiration.

Yet another recent study investigated the differences of KO and FO administration on hepatic and intestinal gene regulation in a high-fat diet mouse model [31]. The study found that KO and FO differently affected the metabolic regulation of lipid degradation and synthesis. While FO induced a PPARα-response by increasing the expression of genes coding for proteins in the two β-oxidation systems and other lipid metabolizing genes, KO seemed to act as a negative regulator of endogenous cholesterol and FA synthesis and gluconeogenesis and only promoted a weak PPARα activation.

### 2.9. Krill Powder

In addition, when KO is provided in the form of krill powder (KP), in a mixture of lipids and proteins (approximately 50% lipids and 40% proteins, Table 1), it has been shown to influence the hepatic levels of inflammatory markers. When transgenic hTNFα mice were fed a high-fat diet with or without KP (6.4% lipids and 4.3% protein, w/w) for 6 weeks, hepatic IL-2 and endogenous TNFα were significantly reduced by the KP treatment only [38]. The plasma cytokines investigated were, however, not affected by KP. The plasma lipids, non-esterified fatty acids (NEFA), PLs and total cholesterol were all significantly decreased in the KP group. TAG levels were also significantly reduced in both plasma and liver in the mice given KP as compared to the control group. These results are consistent with the finding that hepatic expression of genes involved in lipogenesis and glycerolipid synthesis were down-regulated.

The effects of KP on hepatic gene expression have also been investigated in CBA/J mice [41]. Mice were fed either a low-fat control diet or a 3% (w/w) KP low-fat diet for 3 months. While metabolic pathways for β-oxidation, glucose metabolism and amino acid catabolism were down-regulated, an up-regulation of the mitochondrial electron transport chain pathway could be observed. This may indicate increased energy preservation in the KP group during low-calorie intake. In total, KP modulated a high number of pathways, even more so than observed for KO [37], most likely due to the presence of amino acids. The down-regulation of amino acid catabolism was not shown for KO diets and might imply that KP has additional benefits compared to KO alone. The effect of KP on various metabolic pathways is compared with those of KO and FO in Table 3.

**Table 3:** Metabolic changes in mice receiving either a low or high fat diet with inclusion of krill oil or krill powder (summary from [26,37,38]). Adapted with permission from [41].

Metabolic Pathway	Krill Oil		Krill Powder	
	High-fat	Low-fat	High-fat	Low-fat
Lipid synthesis	↓	↓	↓	-
Cholesterol metabolism	↓	↓	↓	-
β-oxidation	↑	↓	↑	↓
Mitochondrial respiration	?	↑	?	↑
Amino acid catabolism	?	-	?	↓
Glucose synthesis	↓	↓	↓	↓

(↓) Reduction in pathway or gene expression/enzyme activity. (↑) Increase in pathway or gene expression/enzyme activity. (-) No change in pathway or gene expression/enzyme activity. (?) No data available.

A more concentrated Antarctic krill protein product (around 8% lipids and 78% proteins, Table 1) can also be obtained by applying an isoelectric solubilization/precipitation method [57]. This leads to a safe to consume low dose omega-3 krill protein concentrate (KPC) [39], where DHA is mainly found in the TAG form. Nevertheless, after 4 weeks of feeding a 10% KPC diet to young female Sprague-Dawley rats, EPA and DHA tissue concentrations were significantly increased when compared to a control group [42]. Moreover, effects of KPC feeding to rats on renal and bone health were investigated in another 4-week study [40]. While no effects on bone mass and strength could be observed, early renal injury was prevented by KPC consumption compared to rats fed casein alone.

## 3. Discussion

Growing pressure on fish stocks increases the demand for new sustainable *n*-3 PUFA sources. Krill could be an alternative source, providing *n*-3 PUFAs in the form of KO, KP and KPC. With the added benefit of not only supplying *n*-3 PUFAs, but also the choline component of the PLs, krill products present a wide potential as seen in various animal experiments outlined in this review. However, how much of the animal data can be extrapolated to humans remains to be seen. It has been cautioned that when it comes to PPARα activation, not all results from rodent studies can be directly applied to human health, in particular when predicting tissue-specific effects [58] and effects on lipoprotein metabolism [59].

While both KO and KP administration to mice affected a similar amount of genes (around 4880 genes), in comparison FO seemed less bioactive by affecting only 192 genes at similar doses of EPA and DHA (0.46%, 0.50%, and 0.54%, w/w, in the KP, KO and FO diet, respectively) [37,41]. Differential gene expression profiles might be explained by different hepatic levels of EPA and DHA when given in PL *versus* TAG form. Which exact mechanisms lie behind different *n*-3 tissue uptake efficiencies are however unknown, but FA integration into different blood pools might be one factor that determines tissue distribution [60].

In the animal studies described here, lowering of plasma lipids can be observed with all *n*-3 PUFA products, however with different efficiencies and to some extent via different mechanisms. A reduction in PPARα levels and in PPARα-activated gene expression seems to be specific for KO/KP included in a low-fat diet and was not observed with FO [31,37,41]. However, in the context of a high-fat feeding, KO/KP demonstrated increased β-oxidation [24,36,38] and the reduction in β-oxidation on a low-fat diet might be due to improved energy preservation during low-calorie conditions. Lipogenesis and cholesterol production were reduced on the gene level upon KO/KP feeding with both low- and high-fat diets and more so than after FO administration [26,36,37,38]. It is noteworthy that FO led to accumulated liver fat in the gene expression study in mice, whereas KO decreased it [31].

Hepatic fat deposition in non-alcoholic fatty liver disease (NAFLD) is the sum of various metabolic pathways including lipogenesis, β-oxidation, VLDL synthesis/secretion and more. Based on the ability of the *n*-3 PUFAs of KO to positively affect some of these pathways as well as the endocannabinoid system in addition to a potential influence on liver health by the PL/choline part of KO [61], dietary KO might provide also in clinical settings benefits to alleviate the pathogenesis of NAFLD (reviewed in [62]).

Another area of interest that might be worth considering in future studies is the effect of KO and KP on glucose metabolism. While KO/KP led to a significant reduction in the expression of genes involved in glucose metabolism [37,41], FO revealed no such effect [37]. Meta-analysis on subjects with type-2 diabetes or glucose intolerance, confirm these results and show no effect of FO on diabetic parameters such as fasting glucose and insulin [63,64]. Whereas, at least in the few animal studies that have been performed on the effects of KO on glycemic control and fasting insulin, a significant reduction in blood glucose and insulin could be observed [24,30,32]. Assessments of potential differences of KO to FO supplementation on the development of insulin resistance and type-2 diabetes mellitus might therefore be attractive in clinical follow-up studies.

An increased intake of *n*-3 PUFAs has been related to improvements of brain health (e.g., cognitive function, Alzheimer’s disease, depression and schizophrenia [65]). Since KO significantly increased the amount of EPA and DHA in the brain of Zucker rats [28] and improved learning and anti-depressant-like effects have been observed in other animal studies [22,35], also brain health might be an area of future interest for clinical KO research. In this respect, it is noteworthy that KO contains EPA and DHA in a 2:1 ratio, while a recent meta-analysis supported the improvement of primary depression by *n*-3 PUFAs only if the supplements contained more than 60% of EPA [66]. Moreover, the high PC content of KO might achieve a more pronounced DHA brain uptake, since it was shown that Mfsd2a, the main DHA transporter in the blood-brain barrier vessels, preferentially binds to lyso-PC [11].

A specific trait that only supplementation with KP revealed, was the effect on amino acid and protein metabolism in mice fed a low-fat-diet, mainly by reducing the expression of genes implicated in protein and amino acid degradation [41]. A KP diet as a source of high-quality marine proteins could be of particular interest in the context of sports nutrition, but also for combating the loss of muscle mass (sarcopenia) that is often associated with aging.

Frailty in the elderly is characterized by weight loss, fatigue/exhaustion, weakness and mobility impairment [67]. In addition, a reduction in mitochondrial function via reduced oxidative phosphorylation and increased oxidative damage was found to contribute to frailty [68]. In the microarray study in mice, KP mostly increased the expression of genes coding for complex I subunits, but also complex II and cytochrome C subunits were affected [41]. It might therefore be possible for KP to positively influence energy metabolism and oxidative stress. In combination with favoring the preservation of amino acids, KP may help to prevent frailty in the elderly and improve quality of life by ameliorating muscle mass and respiratory fluxes.

## 4. Conclusions

In short, by changing the n-6 to *n*-3 PUFA content of cells and thereby their gene expression profiles, as well as modulating endocannabinoid precursor availabilities, KO and KP might benefit health concerns affecting the liver, kidney, muscle and brain and might help to alleviate metabolic dysfunction and its associated cardiovascular disease risk.

By obviating strong conclusions from animal studies, nevertheless KO and KP have shown potential in animal studies to benefit human health in areas that have not been investigated yet, which warrant further investigations in clinical settings.

## Abbreviations

AEA: *N*-arachidonoyl-ethanolamine
2-AG: 2-arachidonoylglycerol
ALA: α-linolenic acid
ALSAT: aversive light stimulus avoidance test
ARA: arachidonic acid
CIC: mitochondrial citrate carrier
CNS: central nervous system
CVD: cardiovascular disease
DAI: disease activity index
DHA: docosahexaenoic acid
DSS: dextran sulfate sodium
EC: endocannabinoids
EPA: eicosapentaenoic acid
FA: fatty acid
FO: fish oil
HDL: high-density lipoprotein
IMIP: Imipramine
KO: krill oil
LDL: low-density lipoprotein
LPS: lipopolysaccharide
LV: left ventricular
lyso-PC: lysophosphatidylcholine
MI: myocardial infarction
NAFLD: non-alcoholic fatty liver disease
NHANES: national health and nutrition examination survey
NEFA: non-esterified fatty acid
NOAEL: no observed adverse effect level
*n*-3 PUFA: omega-3 polyunsaturated fatty acid
PC: phosphatidylcholine
PE: phosphatidylethanolamine
PL: phospholipid
PPARα: peroxisome proliferator-activated receptor alpha
RME: Reference Memory Error
TAG: triacylglyceride
TNFα: tumor necrosis factor-alpha
WME: Working Memory Error
